# An azoospermic factor gene, *Ddx3y* and its paralog, *Ddx3x* are dispensable in germ cells for male fertility

**DOI:** 10.1262/jrd.2018-145

**Published:** 2019-01-07

**Authors:** Takafumi MATSUMURA, Tsutomu ENDO, Ayako ISOTANI, Masaki OGAWA, Masahito IKAWA

**Affiliations:** 1) Research Institute for Microbial Diseases, Osaka University, Osaka 565-0871, Japan; 2) Graduate School of Pharmaceutical Sciences, Osaka University, Osaka 565-0871, Japan; 3) Immunology Frontier Research Center, Osaka University, Osaka 565-0871, Japan; 4) The Institute of Medical Science, The University of Tokyo, Tokyo 108-8639, Japan; 5) Graduate School of Biological Sciences, Nara Institute of Science and Technology, Nara 630-0192, Japan

**Keywords:** Azoospermia factor region, Chimeric analysis, CRISPR/Cas9, Male infertility, Y chromosome

## Abstract

About 10% of male infertile patients show abnormalities in spermatogenesis. The microdeletion of azoospermia factor a (*AZFa*) region of the Y chromosome is thought to be a cause of spermatogenic failure. However, candidate gene responsible for the spermatogenic failure in *AZFa* deleted patients has not been elucidated yet. Using mice, we explored the function of *Ddx3y*, a strong candidate gene in the *Azfa* region, and *Ddx3x*, a *Ddx3y* paralog on the X chromosome, in spermatogenesis. We first generated *Ddx3y* KO male mice using CRISPR/Cas9 and found that the *Ddx3y* KO male mice show normal spermatogenesis, produce morphologically normal spermatozoa, and sire healthy offspring. Because *Ddx3x* KO males were embryonic lethal, we next generated chimeric mice, which contain *Ddx3x* and *Ddx3y* double KO (dKO) germ cells, and found that the dKO germ cells can differentiate into spermatozoa and transmit their mutant alleles to offspring by normal mating. We conclude that *Ddx3x* and *Ddx3y* are dispensable for spermatogenesis at least in mice. Unlike human, mice have an additional *Ddx3y* paralog *D1pas1*, that has been reported to be essential for spermatogenesis. These findings suggest that human and mouse DDX3 related proteins have distinct differences in their functions.

Recent findings have observed infertility occurring among in one in six pairs of couples in developed countries, with half of the infertility due to male factors [1]. About 10% of male infertile patients are categorized as non-obstructive azoospermia (NOA), which is defined as no sperm in the ejaculate due to failure of spermatogenesis [2]. Artificial reproductive technologies, such as ICSI and ROSI, can be applicable even if a few spermatozoa or haploid spermatids are observed in the testis [3, 4]. When spermatogenesis is arrested at the meiotic stage, or when only the somatic Sertoli cells (but not germ cells) are present in the seminiferous tubules of testes (Sertoli cell-only syndrome: SCOS), an effective treatment is limited. Less than 3% of infertile patients who suffer from meiotic arrest or SCOS can be explained by abnormal hormone secretion (hypogonadotropic hypogonadism), such as low FSH and/or LH, and can be treated by hormonal therapy to promote sperm production [2, 5]. The other molecular mechanisms behind NOA are still unclear.

A microdeletion of the Y chromosome is one of the major genetic factors of NOA. In 1976, Tiepolo and Zuffardi examined chromosomes of six NOA patients and found a novel deletion in the long arm of the Y chromosome (Yq11) [6]. The mutation was believed to be de novo as it was not found in their farther or brothers. In 1996, Vogt *et al.* analyzed Yq11 of 370 male infertile patients and found that 12 of them had different microdeletions [7]. These microdeletions are classified into three subgroups, named Azoospermia Factor (AZF) regions (*AZFa*, *AZFb*, and *AZFc*). The deletion of the *AZFa* region is associated with SCOS, which is the severest symptom [7, 8]. However, a candidate gene responsible for spermatogenic failure in *AZFa* deleted patients has not been elucidated yet.

The human *AZFa* region includes three genes, *USP9Y*, *DDX3Y*, and *UTY* [9,10,11,12]. Like in human, the mouse orthologous genes (*Usp9y*, *Ddx3y*, and *Uty*) locate close to each other, in the short arm of the Y chromosome [13]. In 2006 and 2009, deletion of *USP9Y* gene has been reported in men whose fathers also contained the deletion, suggesting that *USP9Y* is not the causative gene [14, 15]. In addition, the *Uty* knockout (KO) mice generated by TALEN-based genome editing [16, 17] did not show any problems with fertility [18]. Therefore, *DDX3Y* (*Ddx3y* in mice) is thought to be the candidate factor in *AZFa* deletion induced spermatogenic failure [19]. Indeed, *DDX3Y* has a higher mutation rate in SCOS patients than the other two genes in the *AZFa* region [20], and DDX3Y protein localization is restricted only in testes, specifically in spermatogonia and early spermatocytes, albeit with low mRNA expression of *DDX3Y* in various organs [21].

*DDX3Y* has a paralog on the X chromosome, *DDX3X*, and their homology at the amino acid and nucleotide sequence are 91% and 88%, respectively. In mice, homologies between *Ddx3y* and *Ddx3x* in amino acid sequences and nucleotides are 90% and 84%, respectively. DDX3X protein is expressed in the brain, kidney, ovary, and testis [21]. Interestingly, it is suggested that mouse DDX3X protein is localized in germ cells of the testes, including spermatogonia [22]. At present, functions of DDX3Y and DDX3X in spermatogenesis remains unclear.

In this study, we analyzed the functions of *Ddx3y* and *Ddx3x* in spermatogenesis, using mice. We first generated *Ddx3y* KO male mice by CRISPR/Cas9 [23, 24] and analyzed spermatogenesis. Because *Ddx3x* KO males show embryonic lethality, we next generated chimeric mice, which contain spermatogenic cells derived from ES cells mutated for both *Ddx3x* and *Ddx3y*, and analyzed spermatogenesis in the double KO (dKO) ES chimeric mice.

## Materials and Methods

### Animals

All animal experiments were conducted in accordance with the guidelines of “Animal experiment rules” established by the Research Institute for Microbial Diseases, Osaka University, and were approved by the Animal Care and Use Committee of the Research Institute for Microbial Diseases, Osaka University (#Biken-AP-H25-02). B6D2F1, ICR, and C57BL/6NCr mice were purchased from CLEA (Tokyo, Japan) or SLC (Shizuoka, Japan).

### Plasmid construction and genotyping

Construction of sgRNA/CAS9 expressing plasmids, pX330 (#42230, Addgene, Cambridge, MA, USA), were performed by ligating oligos into the BbsI site of each plasmid as described previously [25, 26]. Potential off-target sites were searched using Bowtie software (http://bowtie-bio.sourceforge.net/index.shtml) as described previously [25]. We chose gRNAs whose 14 bases at the 3’ end, plus the NGG site, only matched the target site. The sgRNA target sequences for generating *Ddx3y* KO mice were 5’- GTCTGTGATAAGGACAGTTC -3’ (sgRNA #1) and 5’- TATTTCAGTGATCGTGGAAG -3’ (sgRNA#2). The sgRNA target sequence for generating *Ddx3x* KO mice was 5’- GTGGCAGTGGAAAATGCGCT -3’. The following primer sets were used for genotyping PCR of *Ddx3y*: Forward primer 5’- CATGCCCTCATCTCAATATCCCATAAGGT -3’ and Reverse primer 5’- GGATAGCCATTGTTGGACTAGTTGGACA -3’, The following primer sets were used for genotyping PCR of *Ddx3x*: Forward primer 5’- CCAAGGCTTCTTTATGAGCCGACG -3’ and Reverse primer 5’- CCACCTGCGGGTCACTAATCAAC -3’.

### Pronuclear injection of mouse fertilized eggs

B6D2F1 superovulated females were mated with B6D2F1 males, then fertilized eggs were collected 20 h after hCG. Circular pX330 plasmids were injected into one of the pronuclei at 5 ng/μl [25, 26]. The eggs were cultured in KSOM overnight [27], and the two-cell stage embryos were transferred into the oviducts of pseudopregnant ICR females.

### Genome editing in mouse ES cells and generation of chimeric mice

The EGR-G01 ES cells [28] (1 × 10^3–4^) were seeded on mouse embryonic fibroblasts (MEF) in a 6-well plate and transfected with pX330 (total 1.0 µg) using Lipofectamine LTX & PLUS technology (Thermo Fisher Scientific, MA, USA). A pPGK-puro plasmid (0.1 µg) was co-transfected. After 14–18 h, the cells were selected with puromycin (0.1 µg/ml) for 48 h, then grown for 5 to 6 more days, picked, and transferred onto MEF cells in 96-well plates. After 48–72 h of culture, each ES cell clone was split in duplicate, for freezing, and DNA harvesting. After PCR amplification and direct sequencing, the positive clones were thawed and expanded to analyze their karyotypes. The mutant ES cell clones with normal karyotypes were injected into 8-cell ICR embryos, and the chimeric blastocysts were transplanted into the uteri of 2.5 dpc pseudopregnant females [29].

### cDNA synthesis and RT-PCR

Testes were collected from C57BL/6NCr and Ddx3y KO male mice. These samples were homogenized in TRIzol (Thermo Fisher Scientific). The total RNA was reverse-transcribed to cDNA using SuperScript III First Strand Synthesis System for RT-PCR (Thermo Fisher Scientific). Five ng of cDNA was used for PCR with primer sets and KOD DNA Polymerase (KOD-Fx Neo, TOYOBO, Osaka, Japan). The following primer sets were used for detecting *Ddx3y*: Forward primer 5’- ATGAGTCAAGTGGCAGCGG -3’ and Reverse primer 5’- TCAATTGCCCCACCAGTCAACTGCC -3’.

### Prediction of domain structure

The predicted amino acid sequences of wild-type DDX3Y and DDX3X, or mutant DDX3Y and DDX3X were submitted to SMART web tool (http://smart.embl-heidelberg.de/).

### Morphology analysis of spermatozoa

The spermatozoa collected from cauda epididymis of male mice were incubated in TYH medium [30] for 10 min and dispersed in PBS.

### Morphology analysis of embryos

*Ddx3x* heterozygous females were mated with *Ddx3y* KO males. Mouse embryos were collected at embryonic day 10.5 (E10.5), with the visualization of the copulatory plug considered to be E0.5. Genotype of *Ddx3x* was verified by PCR and DNA sequencing. To determine the sex of embryos, primers amplifying *Uba1* (ubiquitin-like modifier activating enzyme 1) (X chromosome) and *Uba1y* (ubiquitin-like modifier activating enzyme 1) (Y chromosome) were used. The size of the PCR product for *Uba1* or *Uba1y* is 211-bp or 183-bp, respectively. The primers used were 5’-TGGTCTGGACCCAAACGCTGTCCACA -3’and 5’- GGCAGCAGCCATCACATAATCCAGATG -3’.

### Mating test

Male mice were caged with 2 B6D2F1 females for 3 months. The number of delivered pups was counted. Frozen spermatozoa from *Ddx3y* disrupted males (X/Y*^Ddx3y-em2^*) will be available through RIKEN BRC (http://en.brc.riken.jp/index.shtml) and CARD R-BASE (http://cardb.cc.kumamoto-u.ac.jp/transgenic/). The stock ID number of *Ddx3y* KO mouse strain is 09785 (Riken BRC) or 2432 (CARD), respectively.

### Histological analysis of testis

After mating test, males were sacrificed by cervical dislocation following anesthesia. Testes were fixed in 4% paraformaldehyde in PBS and were processed for plastic sectioning using Technovit® 8100 (Mitsui chemicals, Tokyo, Japan) according to the manufacturer's instruction. Briefly, fixed testes were washed in PBS at 4ºC for an hour, dehydrated in acetone at 4ºC for an hour, infiltrated with in mixed solution of Technovit 8100 basic solution and hardener 1 (1.5 ml of basic solution plus 9 mg of hardener 1 per sample) at 4ºC for 2–6 h, and then embedded after adding 50 µl of hardener 2. For analysis of *Ddx3y* KO mouse testes, 5 µm sections were treated with 1% periodic acid for 10 min, followed by treatment with Schiff's reagent (Wako, Osaka, Japan) for 20 min. The sections were stained with Mayer's hematoxylin solution prior to imaging, and observed under a microscope. For analysis of EGFP-labeled spermatozoa in *Ddx3x* and *Ddx3y* dKO ES chimeric mouse testes, 5 µm sections were stained with 65 μM Hoechst 33342 (Life Technologies) in PBS for 5 min, and observed under a fluorescence microscope.

### Statistical analysis

All values are shown as the mean ± SD of at least three independent experiments. Statistical analyses were performed using Student’s *t*-test for comparing the number of pups (litter size) derived from wild-type females mated with wild-type or *Ddx3y* KO males, and using Tukey HSD test for comparing litter size derived from *Ddx3x* heterozygous females mated with *Ddx3y* KO males.

## Results

### Generation of Ddx3y deficient mice

**Fig. 1.**
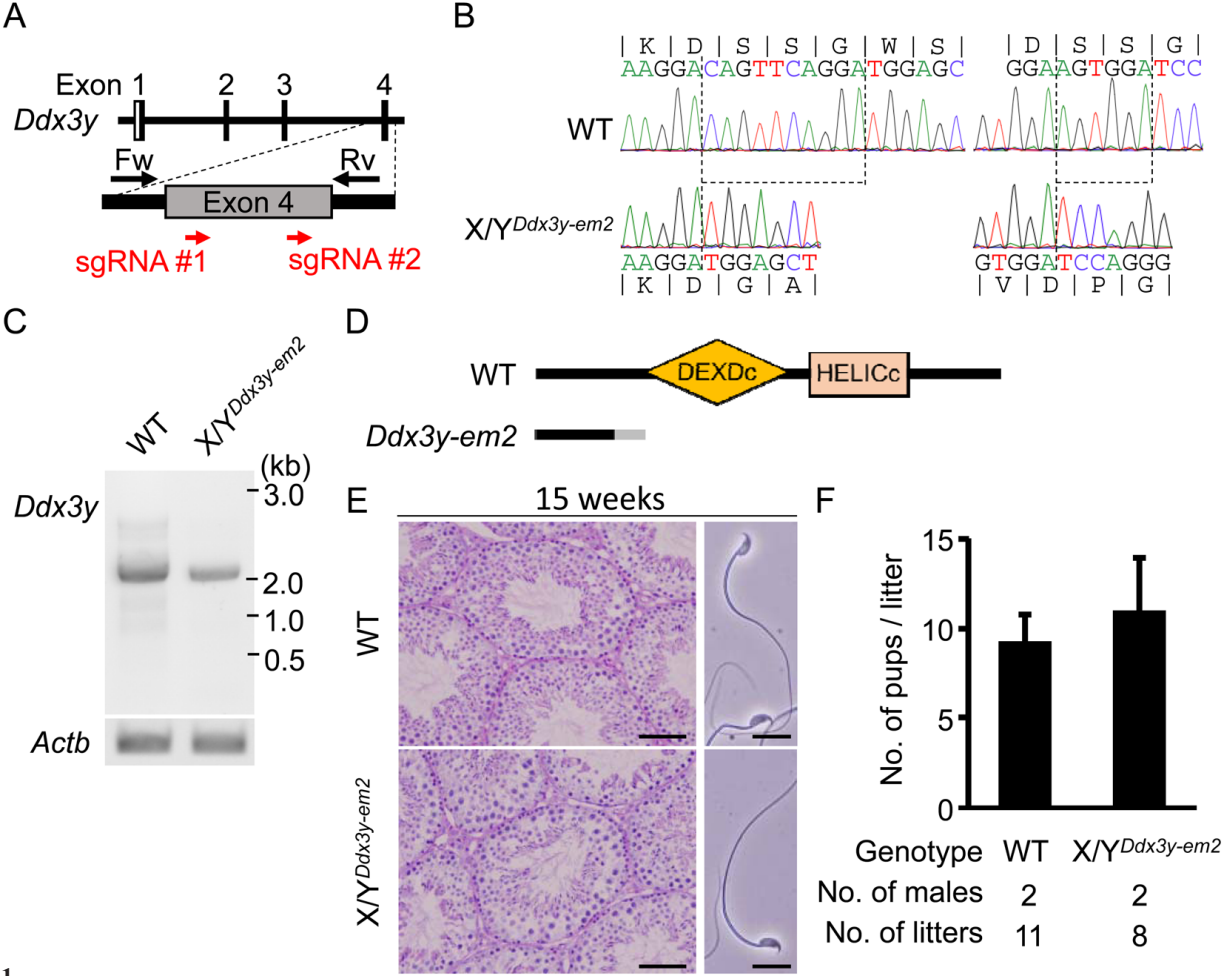
*Ddx3y* deficient male mice have normal spermatozoa and sire offspring. (A) Design of sgRNAs for generating *Ddx3y* KO mice. Red arrows indicate the location of sgRNAs. Black arrows indicate primers used for genotyping. White and gray boxes show untranslated region and coding sequences, respectively. (B) Genomic sequence of wild-type and *Ddx3y* KO (Y*^Ddx3y-em2^*) alleles; 10-bp and 6-bp are deleted (dashed lines). (C) RT-PCR of full length transcripts of *Ddx3y* from wild-type and *Ddx3y* KO testis cDNA. *Actb* (actin beta) was used as control. (D) Predicted protein product of the wild-type and *Ddx3y* KO allele. Orange and pink boxes represent the DEXDc and HELICc RNA-helicase domains respectively. The gray region in *Ddx3y* KO indicates amino acid sequence differing from wild-type. (E) Testicular sections (Left) and sperm morphology (Right) of 15-week-old wild-type and *Ddx3y* KO mice. Testicular sections were stained with hematoxylin and PAS. Scale bar: 50 µm (Left). Sperm morphology of wild-type and *Ddx3y* KO. Scale bars: 20 µm (Right). (F) Average litter size derived from wild-type and *Ddx3y* KO males. Error bars represent standard deviation (SD).

In order to generate *Ddx3y* KO mice, we performed genome editing by pronuclear injection of fertilized eggs using the CRISPR/Cas9 system. Mouse *Ddx3y* consists of 17 exons. We designed 2 single guide RNAs (sgRNAs) targeting the fourth exon of *Ddx3y* (#1 and #2) (Fig. 1A

). We perfomed pronuclear injection of a combination of pX330s expressing human codon-optimized Cas9 and sgRNA #1 or #2. Mutations of *Ddx3y* in founder mice (F0) were confirmed by PCR and sequencing. From 94 injected eggs, 2 out of 5 male offspring (40%) had mutations in *Ddx3y*. One male had two small indels, 10-bp and 6-bp at #1 and #2 target sites, respectively, that resulted in the frame shift (X/Y*^Ddx3y- em2^*) (Fig. 1B). The 8-week-old X/Y*^Ddx3y-em2^* founder male was mated with 2 of wild-type BDF1 females, and produced male offspring (F1) to establish the *Ddx3y* mutant line for subsequent analysis.

To predict whether any potential DDX3Y protein generated in the mutant is functional, we next amplified the entire ORF region of the mutated *Ddx3y* by RT-PCR, sequenced the cDNA, and examined potential reading frames. As expected, the *Ddx3y* band from cDNA of X/Y*^Ddx3y-em2^* mice showed a similar size to that in wild-type mice (Fig. 1C), without any other detectable bands. We confirmed the cDNA from the mutant sequence showed a frameshift mutation with a stop codon before the RNA helicase domain (DEXDc) (Fig. 1D and Supplementary Fig. 1: online only), suggesting that only non-functional protein without an RNA helicase domain can be translated. Therefore, X/Y*^Ddx3y- em2^* males were considered KO mice.

### Ddx3y deficient male mice have normal spermatozoa and sire offspring

There is a possibility that the founder *Ddx3y* KO mice (F0) generated by pronuclear injection were genotypically mosaic [31]. Thus, we used the male offspring (F1) for phenotypic analysis. To analyze spermatogenesis in *Ddx3y* KO mice (F1), the testis histology was observed by Hematoxylin-PAS staining. *Ddx3y* KO males showed no abnormality in spermatogenesis at 15 weeks of age (Fig. 1E left), suggesting that *Ddx3y* is not required for spermatogenesis or the maintenance of spermatogonial stem cells. We next collected spermatozoa from cauda epididymis of *Ddx3y* KO mice and found that the KO spermatozoa showed normal morphology (Fig. 1E right). Indeed, the litter size derived from *Ddx3y* KO males was comparable to that from WT males (Fig. 1F).

### Ddx3x is required for development

**Fig. 2.**
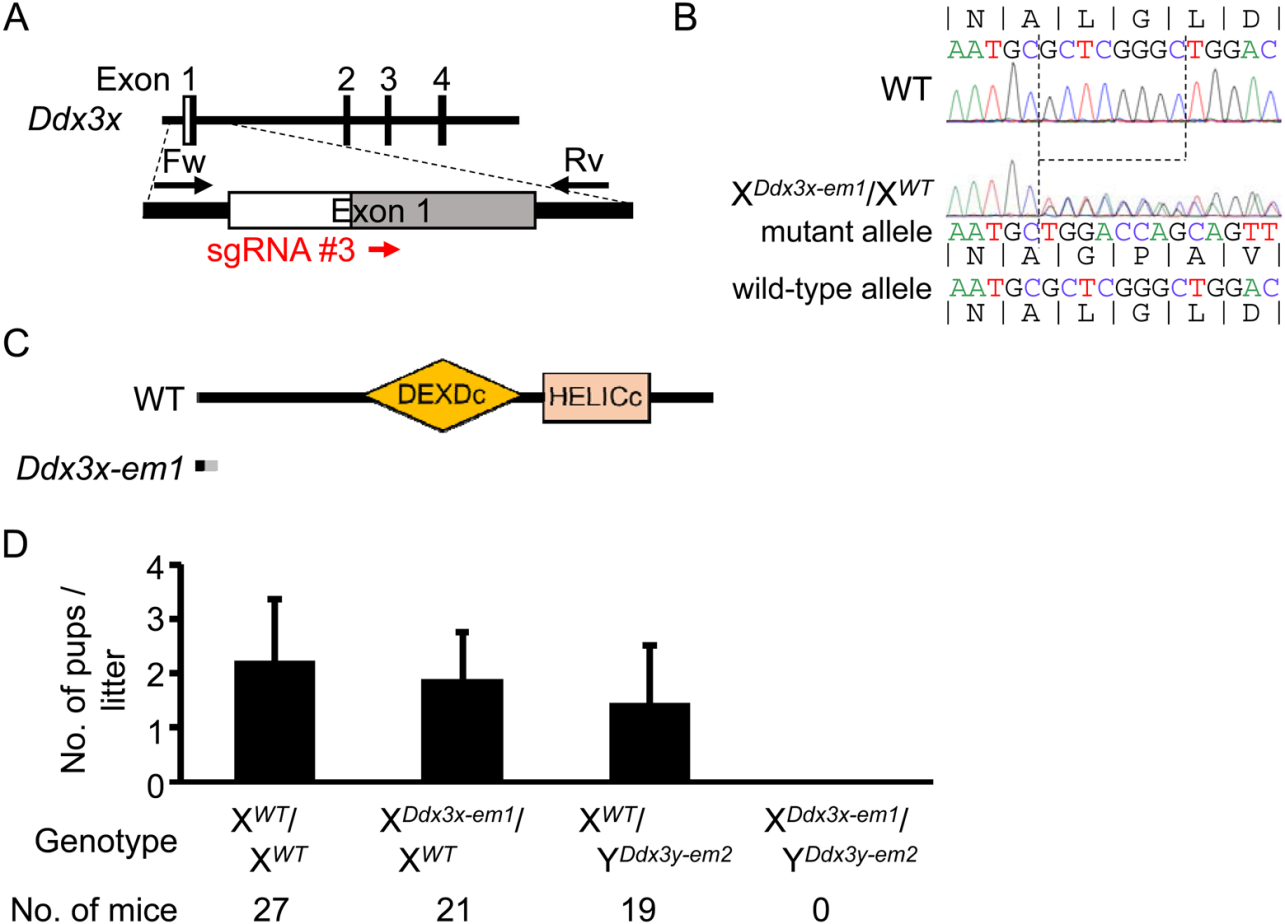
*Ddx3x* and *Ddx3y* double KO mice are embryonic lethal. (A) Design of the sgRNA for generating *Ddx3x* KO mice. Red arrows indicate the location of the sgRNA. Black arrows indicate primers for genotyping. White and gray boxes show untranslated region and coding sequences, respectively. (B) Genomic sequence of wild-type (X*^WT^*) and *Ddx3x* KO (X*^Ddx3x-em1^*) alleles; 8-bp is deleted (dashed lines). (C) Predicted protein product of the wild-type and *Ddx3x* KO allele. Orange and pink boxes represent the DEXDc and HELICc RNA-helicase domains respectively. The gray region in *Ddx3x* KO indicates amino acid sequence differing from wild-type. (D) Average litter size delivered from *Ddx3x* heterozygous mutant females mated with *Ddx3y* hemizygous mutant males. Error bars represent SD.

Mouse *Ddx3x* also consists of 17 exons. We designed sgRNA #3 targeting the first exon of *Ddx3x* (Fig. 2A

) and performed pronuclear injection of pX330s targeting *Ddx3y* (#2) and *Ddx3x* (#3) simultaneously. From 119 injected eggs, 2 out of 24 offspring (8.3%), a male and a female, had a mutation in *Ddx3x*. The male had an in-frame mutation (12-bp deletion) in *Ddx3x* and no mutation in *Ddx3y* (X*^Ddx3x-em1^*/Y*^WT^*). The female had a frame shift mutation (8-bp deletion) in one allele of *Ddx3x* (X*^Ddx3x- em1^*/X*^WT^*), and produced female offspring (F1) with the same mutant allele (Fig. 2B). Sequencing of the allele identified a premature stop codon before the DEXDc sequences (Fig. 2C and Supplementary Fig. 2A: online only), suggesting that only a non-functional protein without an RNA helicase domain can be translated. The F1 X*^Ddx3x- em1^*/X*^WT^* females were mated with X*^WT^*/Y*^Ddx3y- em2^* males, but dKO males (X*^Ddx3x-em1^*/Y*^Ddx3y- em2^*) were never obtained (Fig. 2D), suggesting the importance of *Ddx3x* in mouse embryonic development. To determine whether *Ddx3x* is important for male embryonic development, we analyzed embryos at E10.5 and found only one dKO male out of 34 embryos (2.9%), which was lower than the Mendelian ratio (25.0%), and the dKO male was also smaller than wild-type embryos (Supplementary Fig. 2B and 2C). These results are consistent with a recent report showing that *Ddx3x* KO males exhibit embryonic lethality because of abnormal embryogenesis and placental dysfunction [32].

### Establishing Ddx3x and Ddx3y double KO ES cells using CRISPR/Cas9

**Fig. 3.**
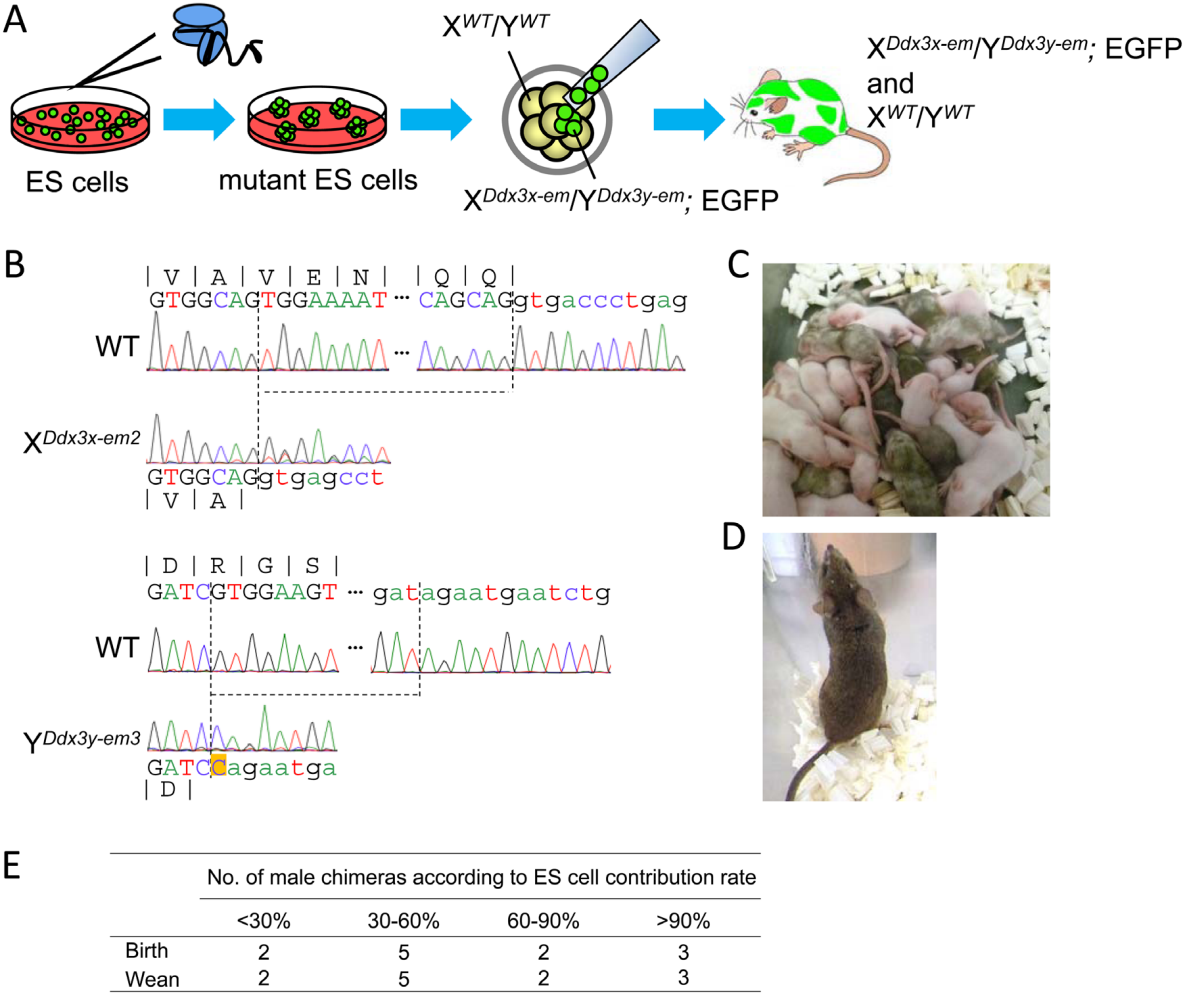
Establishment of the *Ddx3x* and *Ddx3y* double KO ES cell clones using CRISPR/Cas9 system. (A) Schematic of chimeric male mice generation containing *Ddx3x* and *Ddx3y* double KO cells (EGFP positive). The image is adapted from ref. [29]. (B) (Upper region) Genomic sequence of wild-type and *Ddx3x* KO (X*^Ddx3x-em2^*) alleles; 29-bp is deleted (dashed lines). (Lower region) Genomic sequences of wild-type and *Ddx3y* KO (Y*^Ddx3y-em3^*) alleles; 125-bp is deleted (dashed lines). Orange box in *Ddx3y*-*em3* allele indicates 1-bp (cytosine) insertion. Exons are in upper case letters and introns in lower case. (C) Male and female chimeric pups and ICR pups delivered from foster mothers. ES cell contribution is judged by coat color of mice: agouti (ES cells) and white (ICR). (D) Adult male chimera with more than 90% of coat color derived from ES cells. (E) Viability of male chimeras from various ES cell contribution rates. All 13 chimeras are viable after 4 weeks of age (wean) with a high contribution of ES cells.

In order to bypass the embryonic lethality of *Ddx3x* KO males, we utilized chimeric analysis (Fig. 3A

). In 2016, we reported that chimeric mice derived from ES cells carrying a biallelic mutation in a lethal gene can overcome embryonic lethality due to the presence of wild-type cells [29]. This chimeric approach is a useful tool to analyze the function of lethal genes in spermatogenesis [29, 33]. We first transfected ES cells with pX330s, targeting *Ddx3x* (#3) and *Ddx3y* (#2) (Fig. 1A and 2A), into EGR-G01 ES cells [28]. The EGR-G01 ES cells were used as they ubiquitously express EGFP in the cytoplasm of all cell types and the acrosome of spermatozoa [28]. All ES cell clones (8/8) had mutations in both *Ddx3x* and *Ddx3y*. To generate chimeric mice, we used one ES cell line, which had frame shift mutations in both *Ddx3x* (29-bp deletion) and *Ddx3y* (125-bp deletion and 1-bp insertion) (X*^Ddx3x-em2^*/Y*^Ddx3y- em3^*) (Fig. 3B). The obtained chimeric male mice were all viable (13/13) even though ES cell contribution rate, judged by coat color, was 90% or higher (Fig. 3C–E). We decided to use the mice showing high contribution rate (> 90%) for the analysis of spermatogenesis.

### Ddx3x and Ddx3y double KO germ cells in chimeric males differentiate into spermatozoa and transmit their mutant alleles to offspring

**Fig. 4.**
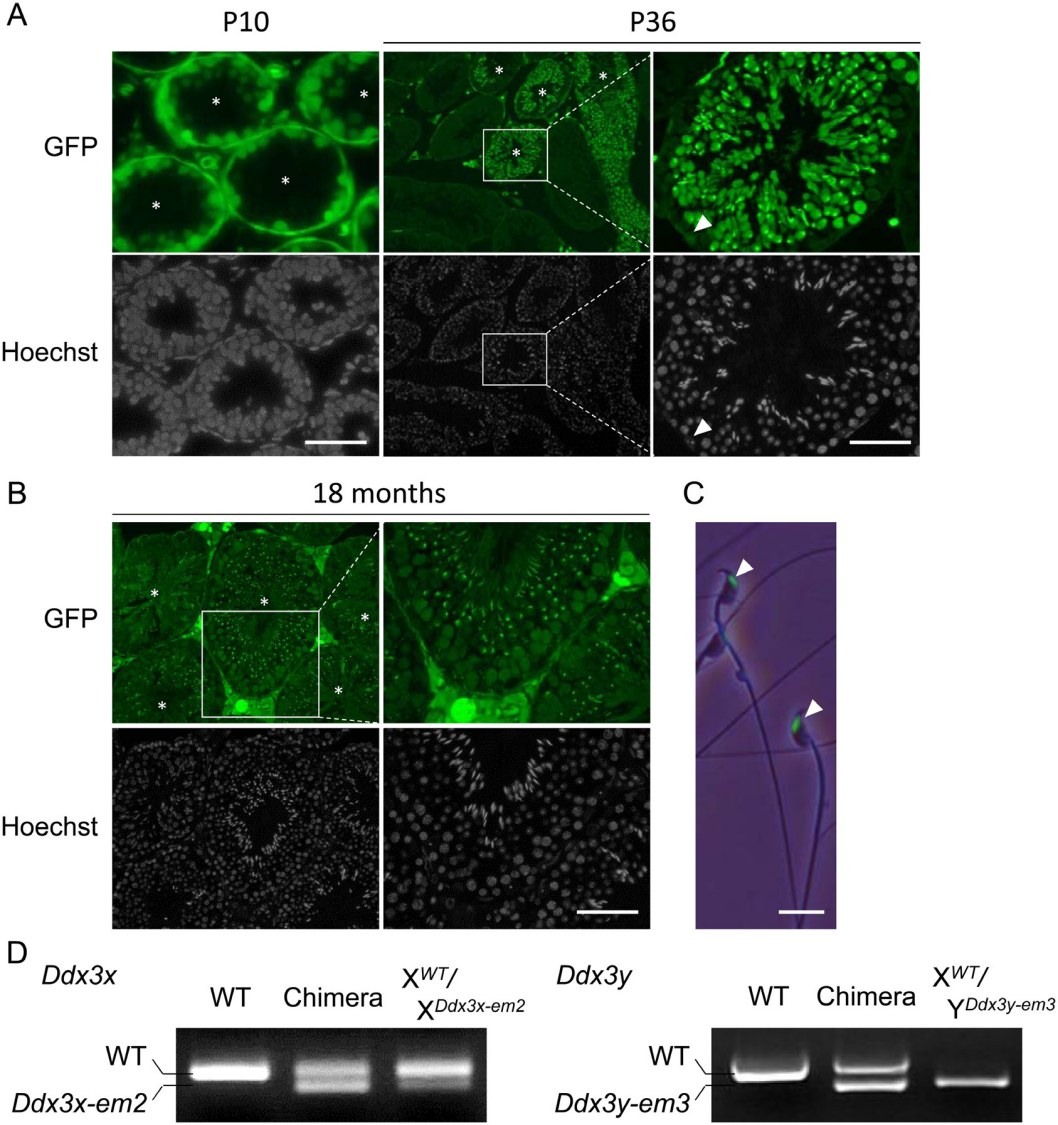
*Ddx3x* and *Ddx3y* double KO germ cells in chimeric males produce functional spermatozoa. (A) (Left) Testicular sections of *Ddx3x* and *Ddx3y* double KO ES chimeric males at postnatal day 10 (P10). Asterisks (*) indicate seminiferous tubules which contain ES cell contributed germ cells (EGFP positive). Scale bar: 50 µm. (Middle) Testicular sections of *Ddx3x* and *Ddx3y* double KO ES chimeric males at P36. Asterisks (*) indicate seminiferous tubules which contain ES cell contributed germ cells (EGFP positive). (Right) Magnified images of middle panel. White arrowhead indicates a Sertoli cell labeled with EGFP. Scale bars: 50 µm. (B) Testicular sections of double KO ES chimeric males at 18 months of age. Asterisks (*) indicate seminiferous tubules which contain ES cell contributed germ cells (EGFP positive). Scale bar: 50 µm. (C) Sperm morphology of double KO ES chimeric males. White arrowheads indicate spermatozoa derived from ES cells (EGFP positive heads of spermatozoa). Scale bar: 10 µm. (D) Genotyping of F1 females (Left; X*^WT^/*X*^Ddx3x-em2^*) and males (Right; X*^WT^/*Y*^Ddx3y-em3^*) derived from chimeras. *Ddx3x* KO (29-bp deletion) band is slightly smaller than the wild-type. *Ddx3y* KO (125-bp deletion and 1-bp insertion) band is slightly smaller than the wild-type.

To confirm the contribution of *Ddx3x and Ddx3y* dKO ES cells to the germ lineage, we generated chimeic mice with the dKO ES clone and examined testicular sections at postnatal day 10 (P10). Germ cells were labeled with EGFP, indicating the contribution of dKO ES cells to germ cells (Fig. 4A

). At P36, spermatids, spermatozoa, and Sertoli cells were labeled with EGFP in some seminiferous tubules (Fig. 4A). Spermatozoa labeled with EGFP were also present in 18-month-old chimeric mouse testes (Fig. 4B), indicating that *Ddx3x* and *Ddx3y* is not cell autonomously required for spermatogenesis or maintenance of spermatogonial stem cells. No morphological abnormalities were observed in EGFP-labeled spermatozoa collected from cauda epididymis of 8-week-old chimeric mice (Fig. 4C). Finally, mating these chimeric male mice with wild-type female mice showed successful transmission of *Ddx3x* or *Ddx3y* mutant allele to the next generation of female or male mice, respectively (Fig. 4D).

## Discussion

We investigated the function of mouse *Ddx3y*, one of three genes present in the *Azfa* region, and mouse *Ddx3x*, an X chromosomal gene homologous to *Ddx3y*, in spermatogenesis. It has been difficult to knockout Y chromosome related genes by conventional gene targeting with homologous recombination in ES cells because the Y chromosome is composed of a variety of repetitive DNA sequences [34, 35]. Recently developed genome editing techniques, such as TALEN and CRISPR/Cas9 systems enable us to target genes with specific 20–30 bp short sequences. Here, we succeeded in generating *Ddx3y* KO male mice by using CRISPR/Cas9 system. We found that *Ddx3y* KO male mice show no abnormality in spermatogenesis, produce normal morphology of spermatozoa, and sire offspring. We conclude that *Ddx3y* is dispensable for spermatogenesis at least in mice. In addition, because *Ddx3x* KO males were found to be embryonic lethal, we generated chimeric mice, which contain *Ddx3x* and *Ddx3y* dKO germ cells, and found that these dKO germ cells can differentiate into spermatozoa and transmit their mutant alleles to offspring. Because both *Ddx3x* and *Ddx3y* mRNAs are expressed in germ cells [36] and are predicted as non-secretory proteins lacking signal peptides, it is unlikely that wild-type germ or somatic cells present in chimeric mice compensate for the function of *Ddx3x* and *Ddx3y* in dKO germ cells. Thus, we conclude that the both *Ddx3x* and *Ddx3y* are not required for spermatogenesis at least in mice.

Recently it was reported that when human iPS cell lines with *AZFa* deletions were xenotransplanted to mouse testes, spermatogenic failure of the derived germ cell-like cells were partially rescued by introduction of DDX3Y [37]. This suggests that DDX3Y is functional during spermatogenesis in human. Interestingly, in mice, *Ddx3y* has an autosomal paralog on chromosome 1, *D1pas1*, which is thought to be pseudogene in human [38, 39]. *D1pas1* has 87% and 80% homology with *Ddx3y* at the amino acid and nucleotide sequence level respectively, respectively, and the mRNA expression is testis specific [36]. Based on our finding, we hypothesize that *D1pas1* has similar functions to *Ddx3y*, and thus can mask the phenotype of *Ddx3y* KO mice. Consistent with our hypothesis, it has been recently reported that spermatogenic failure is observed in *D1pas1* KO mice, indicating the requirement of *D1pas1* for spermatogenesis [40]. Because we showed no spermatogenic failure in *Ddx3y* KO mice, *D1pas1* might overcome *Ddx3y* deficiency. However, *D1pas1* mRNA is expressed in spermatocytes, but not in spermatogonia [36], and *D1pas1* KO germ cells arrest in late pachytene spermatocytes [40], indicating that the spermatogenic failure in *D1pas1* KO mice is milder than that in *AZFa*-deleted SCOS patients. These reports suggest that mouse *D1pas1* alone is not sufficient to function as a substitute for human *AZFa* genes. Thus, it will be useful to conduct studies to determine whether *Ddx3y* and *D1pas1* compensate for each other in mice by generating *Ddx3y* and *D1pas1* dKO mice.

## Supplementary

Supplement Figures
