# Supplementary material for: An azoospermic factor gene, Ddx3y and its paralog, Ddx3x are dispensable in germ cells for male fertility
Source: J Reprod Dev. 2019 Jan 7;65(2):121–8. doi: 10.1262/jrd.2018-145 (PMC6473106; doi:10.1262/jrd.2018-145)
Supplement: Supplement Figures [file jrd-65-121-s001.pdf]

Supplementary figure. 1 (Matsumura *et al.*)

```
         WT MSQVAAESTAGLDQQFVGLDLKSSDNQNGGGNTESKGRYIPPHLRNRETSKGVCDKDSSGWSCSKDKDAYSSFGSRDSRG 80
Ddx3y-em2 MSQVAAESTAGLDQQFVGLDLKSSDNQNGGGNTESKGRYIPPHLRNRETSKGVCDKDGAVVKIKMPTAVLDLVIPEGSPI 80

         WT KPNYFSDRGSGSRGRFDDHGRNDYDGIGGRDRTGFGKFERSGHSRWSDRSDEDDWSKPLPPSERLEQELFSGGNTGINFE 160
Ddx3y-em2 ISVIVDPGEGSMIMVEMTMMVLVVVTELDLANLNGVVIVVGVTDQMKMTGQNHFHQVNA--------------------- 139

         WT KYDDIPVEATGNNCPPHIENFSDIEMGEIIMGNIELTRYTRPTPVQKHAIPIIKEKRDLMACAQTGSGKTAAFLLPILSQ 240
Ddx3y-em2 --------------------------------------------------------------------------------

         WT IYTDGPGEALKAMKENGRYGRRKQYPISLVLAPTRELAVQIYEEARKFSYRSRVRPCVVYGGADTVQQIRDLERGCHLLV 320
Ddx3y-em2 --------------------------------------------------------------------------------

         WT ATPGRLVDMMERGKIGLDFCKYLVLDEADRMLDMGFEPQIRRIVEQDTMPPKGVRHTMMFSATFPKEIQMLARDFLDEYI 400
Ddx3y-em2 --------------------------------------------------------------------------------

         WT FLAVGRVGSTSENITQKVVWVEELDKRSFLLDLLNATGKDSLTLVFVETKKGADSLENFLFQERYACTSIHGDRSQKDRE 480
Ddx3y-em2 --------------------------------------------------------------------------------

         WT EALHQFRSGRKPILVATAVAARGLDISNVKHVINFDLPSDIEEYVHRIGRTGRVGNLGLATSFFNERNLNITKDLLDLLV 560
Ddx3y-em2 --------------------------------------------------------------------------------

         WT EAKQEVPSWLESMAYEHHYKGSSRGRSKSRFSGGFGARDYRQSSGSANAGFNSNRANSSRSSGSSHNRGFGGGGYGGFYN 640
Ddx3y-em2 --------------------------------------------------------------------------------

         WT NDGYGGNYNSQAVDWWGN                                                               658
Ddx3y-em2 ------------------
```

Fig. S1. Predicted protein product of the *Ddx3y* KO allele
Amino acid sequence from the cDNA sequence of wild-type and *Ddx3y* KO (*Ddx3y-em2*) allele. Gray boxes indicate amino acids differing from the wild-type sequence. Orange bar: DEXDc domain. Pink bar: HELICc domain.

Supplementary figure. 2 (Matsumura *et al*.)

A

```
WT          MSHVAVENALGLDQQFAGLDLNSSDNQSGGSTASKGRYIPPHLRNREATKGFYDKDSSGWSSSKDKDAYSSFGSRGDSRG 80
Ddx3x-em1   MSHVAVENAGPAVCWPRPELFR---------------------------------------------------------- 22

WT          KSSFFGDRGSGSRGRFDDRGRGDYDGIGGRGDRSGFGKFERGGNSRWCDKSDEDDWSKPLPPSERLEQELFSGGNTGINF 160
Ddx3x-em1   --------------------------------------------------------------------------------

WT          EKYDDIPVEATGNNCPPHIESFSDVEMGEIIMGNIELTRYTRPTPVQKHAIPIIKEKRDLMACAQTGSGKTAAFLLPILS 240
Ddx3x-em1   --------------------------------------------------------------------------------

WT          QIYADGPGEALRAMKENGRYGRRKQYPISLVLAPTRELAVQIYEEARKFSYRSRVRPCVVYGGAEIGQQIRDLERGCHLL 320
Ddx3x-em1   --------------------------------------------------------------------------------

WT          VATPGRLVDMMERGKIGLDFCKYLVLDEADRMLDMGFEPQIRRIVEQDTMPPKGVRHTMMFSATFPKEIQMLARDFLDEY 400
Ddx3x-em1   --------------------------------------------------------------------------------

WT          IFLAVGRVGSTSENITQKVVWVEEIDKRSFLLDLLNATGKDSLTLVFVETKKGADSLEDFLYHEGYACTSIHGDRSQRDR 480
Ddx3x-em1   --------------------------------------------------------------------------------

WT          EEALHQFRSGKSPILVATAVAARGLDISNVKHVINFDLPSDIEEYVHRIGRTGRVGNLGLATSFFNERNINITKDLLDLL 560
Ddx3x-em1   --------------------------------------------------------------------------------

WT          VEAKQEVPSWLENMAFEHHYKGSSRGRSKSSRFSGGFGARDYRQSSGASSSSFSSSRASSSRSGGGGHGGSRGFGGGGYG 640
Ddx3x-em1   --------------------------------------------------------------------------------

WT          GFYNSDGYGGNYNSQGVDWWGN                                                           662
Ddx3x-em1   ----------------------
```

B

No. of females	No. of total embryos	No. of embryos			
		X^{WT}/X^{WT}	$X^{Ddx3x\text{-}em1}/X^{WT}$	$X^{WT}/Y^{Ddx3y\text{-}em2}$	$X^{Ddx3x\text{-}em1}/Y^{Ddx3y\text{-}em2}$
3	34	17 (50.0%)	4 (11.7%)	12 (35.2%)	1 (2.9%)

C

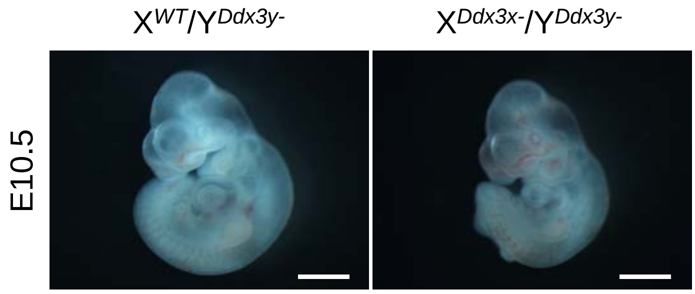

Fig. S2. Embryonic development of *Ddx3x* and *Ddx3y* double KO mice

(A) Amino acid sequence is predicted from genomic sequence of the wild-type and *Ddx3x* KO (*Ddx3x-em1*) allele. Gray boxes indicate amino acids differing from wild-type. Orange bar: DEXDc domain. Pink bar: HELICc domain.

(B) Genotypes of embryos at embryonic day 10.5 (E10.5) derived from *Ddx3x* heterozygous females ($X^{Ddx3x\text{-}em1}/X^{WT}$) and *Ddx3y* hemizygous males ($X^{WT}/Y^{Ddx3y\text{-}em2}$). Number of embryos per total embryos (%) are in brackets.

(C) E10.5 embryos of $X^{WT}/Y^{Ddx3y\text{-}em2}$ and $X^{Ddx3x\text{-}em1}/Y^{Ddx3y\text{-}em2}$. Scale bars: 1 mm.
